# High vs low trait primary psychopathy in males: Differences in cardiac responses to emotional film clips

**DOI:** 10.1192/j.eurpsy.2021.1185

**Published:** 2021-08-13

**Authors:** F. Fusina, A. Angrilli

**Affiliations:** 1 Padova Neuroscience Center, Università degli Studi di Padova, Padova, Italy; 2 Department Of General Psychology, Università degli Studi di Padova, Padova, Italy; 3 Institute Of Neuroscience, National Research Council (CNR), Padova, Italy

**Keywords:** psychophysiology, psychopathy, heart rate variability, emotion

## Abstract

**Introduction:**

Primary psychopathy, although not included in DSM-5, is a personality trait characterized by callousness, unemotionality and a low sensitivity to anxiety and fear. From a psychophysiological standpoint, individuals with this trait exhibit a number of alterations, most notably lower heart rate at rest and lower heart rate variability (HRV).

**Objectives:**

We investigated the relationship between primary psychopathy and heart rate dynamics in response to emotional stimuli in a healthy community sample. In the high psychopathy participants we expected to find lower HRV and a general lower cardiovascular responsiveness to aversive emotional stimuli.

**Methods:**

The study was carried out on male students with high (HP) and low scores (LP) of primary psychopathy according to Levenson’s LSRP. The stimuli were 15 short movie clips of different emotional content (Erotic, Scenery, Neutral, Compassion and Fear), lasting 2 minutes each and presented during ECG recording. Mean heart rate (HR) and HRV were analyzed.

**Results:**

Concerning HR, a Category by Group interaction revealed that participants in the HP group did not differentiate among emotional movie clips, whereas those in the LP group manifested significant reduced HR to Fear and Scenery compared to the other clips. Concerning HRV, the main Group effect showed in HP participants a lower HRV than LP subjects, irrespective of the film categories.

**Conclusions:**

Using ecological stimuli is considered more effective in evoking spontaneous emotions, and our results point to a clear alteration of emotional cardiovascular response in high primary psychopathy trait individuals selected from a community sample.

